# Top management team boundary-spanning leadership: Measurement development and its impact on innovative behavior

**DOI:** 10.3389/fpsyg.2022.988771

**Published:** 2022-12-15

**Authors:** Xuemei Liu, Yuan Yu, Xiuwen Zhao, Ningjun Zhang

**Affiliations:** ^1^School of Business Administration, Southwestern University of Finance and Economics, Chengdu, China; ^2^School of Management, Xihua University, Chengdu, China; ^2^School of Law and Public Administration, Yibin University, Yibin, China

**Keywords:** innovative behavior, measurement development, perceived motivational climate, social information processing theory, top management team boundary-spanning leadership

## Abstract

With the advancement of digital economy, organizations around the world need to stretch the boundaries of their strategy, business, and knowledge to gain a competitive advantage and achieve sustainable growth. Although boundary-spanning leadership, with a set of practical tools developed by the Center for Creative Leadership (CCL), has been explored over the past 10 years, the comprehensive understanding of top management team boundary-spanning leadership has still reached no consensus. This research focuses on the concept of top management team boundary-spanning leadership (TMTBSL) and its effect on employees’ innovative behavior. Study 1 comprises the concept of TMTBSL and the measurement of its development. The classical grounded theory was used to analyze biographical texts and in-depth interview data from local Chinese organizations. We developed a 5-dimension scale with14 items for TMTBSL. In Study 2, we empirically examined the impact of TMTBSL on employees’ innovative behavior. The results demonstrate that TMTBSL can promote employee innovative behavior through perceived motivational climate. The theoretical and practical implications are also outlined.

## Introduction

The advent of the digital economy suggests that no organization can preserve with its traditional management approach unchanged. What matters most in organizations amid the trends of change is the top management team (TMT): whether it can quickly scout and recognize transformation signals, seize fleeting opportunities, and impel the organization to adjust to a “new course” (Ancona and Caldwell, 1992). The capability of TMT to promote direction, alignment, commitment, breaking and across boundaries in the service of a greater vision is key to organizations today, which has been conceptualized as boundary-spanning leadership (BSL; Ernst and Yip, 2009; Ernst and Chrobot-Mason, 2011; Cross et al., 2013). Due to the critical role of TMT in organizations, boundary-spanning leadership in TMT is vital to break boundaries in and out of the organization. Previous studies have described the value of TMT boundary-spanning as that of external integration which benefits from specific boundary roles and positions (Collins and Clark, 2003; Ferguson et al., 2019); meanwhile, the Center for Creative Leadership (CCL) has given BSL strategies such as managing boundaries, forging common ground, and discovering new frontiers (Lee et al., 2014). However, few studies have discussed how BSL works inside the organization. In short, we believe that top management team boundary-spanning leadership (TMTBSL) is not just a type of boundary behavior, but some wider influences to organizations to stretch the boundaries of their strategy, business, and knowledge to gain a competitive advantage and achieve sustainable growth.

Top management team refers to the group that sets the organizational strategy, makes strategic decisions, and serves as the main link outside the organization (Hambrick, 1994; Oh et al., 2004). Therefore, TMTs are not only the “resource integrator” at the boundary of the organization, but also the impellers and leaders who break the “old world order” and build the “new world.” In this research, TMTBSL is defined as the combined influence emerging from organizational strategy decision-making teams to carry out organizational cross-boundary innovation. By definition, TMTBSL is not the simple stack of boundary-spanning leadership capacities of single leaders in TMT (Raes et al., 2007), but the combined influence of leaders and the leadership process.

Existing studies have mainly explained individual-level boundary-spanning leadership and focused on explicit boundaries across organizations or teams (Salem et al., 2018). In fact, however, the boundaries that organizations need to cross involve much more. Evidence has shown that longitudinal boundary (e.g., hierarchy), horizontal boundary (e.g., functions and departments), stakeholders’ boundary (e.g., partners and suppliers), demographic boundary (e.g., gender), and geographical boundary (e.g., region) are the boundaries that organizations must cross for collaborative symbiosis (Ancona and Caldwell, 1992; Ernst and Chrobot-Mason, 2011; Cross et al., 2013). In the organization settings, TMTs may be the most powerful implementers for cross-boundary promotion (O'Reilly et al., 2010; Menz, 2012), as they always make unparalleled contributions to create organizational competitive advantages and interact frequently with outsiders (Geletkanycz and Hambrick, 1997). Nevertheless, due to the uncertainty of successful change, they are also demotivated to change at some point (Georgakakis et al., 2017). Therefore, how to manage the vast majority through the key minority to make cross-boundary promotions and ultimately realize collaborative symbiosis, may be a common challenge for organizations today (Schubert and Tavassoli, 2020; Zhang et al., 2021).

Scholars have paid attention to the importance of overall leadership (Friedrich et al., 2016), and the research on leadership has been expanded from the individual level to the team level (Berson and Avolia, 2004; Carson et al., 2007). Researchers have also noticed that not all leadership enables organizations to strategically adapt to changes and cross the boundaries (Storey, 2005; Simsek et al., 2018). With the rapid development and application of information and communications technology, organizations increasingly need TMTs to hold the proverbial steering wheel to effectively promote boundary-spanning, including some intersecting areas of decision-making. TMT not only makes critical influence on the successful implementation of boundary-spanning strategy within organizations successfully (Wu et al., 2019), but also plays a key role in promoting the boundary-spanning behaviors between organizations (Finkelstein and Hambrick, 1990). A recent study found that TMT boundary-spanning can benefit firm performance based on the structural position of outside directorship and its interaction (Ferguson et al., 2019). Moreover, Ferguson et al. (2019) used one indicator (i.e., outside director) to measure TMTBSL. This research argues that serving as an outside director does not fully match the concept of TMTBSL.

We designed two studies to explore the structural connotation of TMTBSL and its impact on individual innovative behavior. This research makes several theoretical contributions. First, based on the integration of boundary-spanning leadership literature and TMT research, we proposed the concept of TMTBSL and investigated its structural connotation. Second, this study developed a measurement tool of TMTBSL, which contributes to further in-depth empirical analysis. Third, by conducting a field survey, we examined the impact of TMTBSL on individuals’ innovative behavior. We empirically tested the validity of TMTBSL. In sum, this study is at the forefront of understanding the promising concept of TMTBSL.

## Study 1: The dimension and scale development of top management team boundary-spanning leadership

**Figure 1:**
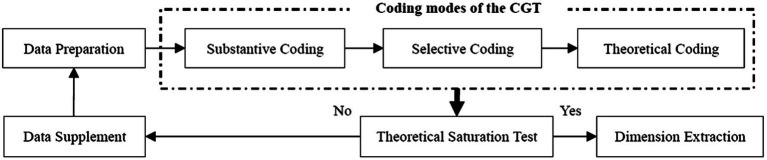
The process of coding and data analysis.

In Study 1, we adopted the classical grounded theory to extract categories from biographies and interview data relevant to the process of boundary-spanning of TMT to construct the item pool (Glaser and Strauss, 1967). The dimensions of TMTBSL were summarized following the coding procedure (Sebastian, 2019), and the items corresponding to each dimension were generated to form the initial scale of TMTBSL. Furthermore, an exploratory factor analysis (EFA) and a confirmatory factor analysis (CFA) were conducted to revise the original scale, which subsequently formals our measurement of TMTBSL (see Figure 1).

### Dimension extraction

The dimension extraction procedure of TMTBSL was followed by Glaser and Strauss (1967) using substantive, selective, and theoretical coding iteratively until reaching theoretical saturation (Sebastian, 2019).

### Study design and samples

According to the ranking of local enterprises in the Hurun China Rich List in recent years^1^, we selected 22 biographies related to 16 TMTs in China as the secondary coding samples. These biographies include various industries such as information technology, agriculture, and chemicals. Additionally, they not only described the process of organizational transformation, development, and critical decisions promoted by TMTs (see Supplementary Appendix S1). In addition, we conducted in-depth interviews with TMTs from six other local enterprises in Southwest China, enabling us to collect first-hand coding samples. In recent years, organizations in Southwest China have witnessed rapid change. For example, the first national supercomputing center in Western China was officially built in September 2020. Hence, the enterprises we interviewed have experienced transboundary innovation based on the industrial ecology of the digital economy. Among the six organizations, one is a listed company, another is an unlisted key enterprise, and the other four are growth small and medium enterprises in a national high-tech industrial park.

In the field research, we conducted semi-structured interviews. The interview outline was formed after a focus group discussion in our research team. As our team members (i.e., three professors and four postgraduates) have ongoing experience in organizational behavior research, we believe that the outline is appropriate and comprehensive. The semi-structured interview outline comprises three parts. The first part is about basic information, such as the type of the organization, the main business. This information helps us to build an accurate understanding of the interviewees and the organization. The second part is mainly about how they recognize and describe the connotations and dimensions of TMTBSL. A sample question is: “What characteristics do you think a TMT needs to have to effectively facilitate crossing boundaries for organization?” The third part is mainly about the influence of TMTBSL. A sample question is: “Can you describe with us some of the key events that the TMT has successfully led and implemented breaking and rebuilding boundaries?”

### Procedure

The coding of biographical texts and field interviews were conducted simultaneously. Four postgraduates participated in the coding of biographical texts using a back-to-back method. Trainings and discussions took place to ensure a common conceptual understanding before we start coding (Bringer et al., 2004). All members of the research team participated in the field interviews. Three professors were responsible for contacting the participated organizations, while the postgraduates were mainly responsible for interview preparation, recording, organizing, and coding. We sent the interview outline to the organization in advance and explained the research purpose. As the field interviews were influenced by the location of the organizations and the schedule of the TMTs, we conducted two rounds of interviews at different times and different locations. The first-round interviews were conducted separately in two enterprises located in southwest China, and the second-round interviews were conducted in four enterprises close to or within a national high-tech industrial park in southwest China. Ultimately, we collected a total of about 300 min of audio recordings and more than 60,000 words of verbatim interviews.

By coding biographical texts and interview data, we summarized 177, 133, 150, and 162 entries, respectively. Then an item pool with 622 entries in total was formed. After discussion and iteration, 154 valid items were obtained by merging similar items. Furthermore, we continued to classify and refine the sub-dimensions and main dimensions through a continuously comparative analysis. Following the reliability test approach (Miles et al., 1994), we extracted 19 sub-dimensions and 5 main dimensions from 154 entries after deleting or modifying sub-dimensions with reliability of less than 0.7. Data collection and coding analysis were terminated until no new information or categories emerged and theoretical saturation had been reached (Glaser, 1978). We also extensively consulted experts and executives in the listed company where we took interviews to revise the coding results (Holton and Walsh, 2017).

### Results

Finally, through the coding of biographical and interview data, we refined five dimensions encompassing the connotation of TMTBSL, namely *Foresight for Boundary-Spanning (FBS)*, *Inspiration for Boundary-Spanning* (IsBS), *Decisiveness for Boundary-Spanning* (DBS), *Integration for Boundary-Spanning* (ItBS), and *Resilience for Boundary-Spanning* (RBS). The corresponding 15 subdimensions and 94 valid items can be seen in Table 1.

**Table 1:** Coding results of TMTBSL.

Dimensions	Sub-dimensions	Concept nodes (Partial)
IsBS		
	Vision inspiring	(a18) establish vision and goals that employees generally agree on; …
	Trait influence	(a12) attract people inside and outside the organization with virtu; …
	Capability attraction	(a1) has the ability to lead and achieve goals; …
DBS		
	Responsibility taking	(a35) take responsibility and has courage to make decisions; …
	Strategy adjustment	(a26) choose the right strategy to promote the healthy development of the organization; …
	Change leading	(a42) lead transformation across boundaries; …
FBS		
	Insight perception	(a55) has a keen sense of timing and seize opportunities; …
	Perspective forecast	(a76) pay attention to the development prospect and grasp the right direction; …
	Boundary-spanning mindset	(a74) has ground-breaking thinking beyond normal; …
ItBS		
	Boundary integration	(a102) reach goals across geographical boundaries; …
	Consensus building	(a98) collaborate across boundaries to achieve common goals; …
	Collaborative promotion	(a89) good at cross-boundary joint innovation; …
RBS		
	Diversity inclusive	(a124) good at creating an inclusive corporate culture; …
	Circumstance adjustment	(a140) adapt to local conditions and carry out cross-boundary operations; …
	Boundary reconstruction	(a143) build strategic alliances and realize resource sharing; …

The author collates the results according to the coding results. The above concept nodes are all translated from Chinese.

FBS, foresight for boundary-spanning; IsBS, inspiration for boundary-spanning; DBS, decisiveness for boundary-spanning; ItBS, integration for boundary-spanning; RBS, resilience boundary-spanning.

#### Foresight for boundary-spanning

FBS refers to the forward-looking vision of the TMT, which can break old-fashioned thinking and establish new cognition for the survival, development, and innovation of the organization. This is supported by the analysis of coding interview transcripts.

*“The second aspect is the vision of the executive team*, *which should be wide and high*. *If you are always doing something at a low level*, *it’s impossible to trans-boundary (A10—boundary-spanning mindset)*. *If you do not have this forward-looking vision*, *you will not be able to see some of the latest and cutting-edge things in the world*, *and you will not be able to predict and make decisions*, *so it’s definitely impossible to have boundary-spanning leadership (A4—perspective forecast)…*. *This is related to the direction and future of the company*. *I think*, *for TMTs*, *these two aspects are very important*.*” (ZQKJ-837)*.

#### Inspiration for boundary-spanning

IsBS refers to the charisma of the TMT, which appeals to the internal members and external stakeholders of the organization to participate in boundary-spanning activities and strive for the common goal. This calling is not the inevitable result of the leader’s legal authority. It reflects the recognition and following of TMT which is the basis for building boundary-spanning consensus. The corresponding interview transcripts are as follows.

*“One is the business direction*, *employees feel that this can be followed*, *worthy of others*, *will be firm in their goal (A17—vision inspiring)*. *We ourselves (TMT members) have the spirit to fight*, *to learn*, *and to belong to this company*. *I think this is the guarantee that the company can go forward*, *survive*, *and achieve high quality goals (A14—trait influence)” (YSTJ-579)*.

*“The first of these is that you have a good grasp of the direction of your industry*, *such as our field*, *which requires an understanding of information technology*, *intelligent manufacturing*, *and (the TMT) must have very good management skills (A11-capability attraction)*.*” (ZQKJ-845)*.

#### Decisiveness for boundary-spanning

Decisiveness for boundary-spanning refers to the leading process in which the TMT is willing and able to take responsibility, act as an agent to make strategic decisions at critical moments, and promote the trans-boundary change of the organization. The rapid changes in and out of the organizations have increased various uncertainties and risk pressures, and also increased the difficulty of decision-making to the TMTs. If they fail to make timely strategic decisions, the organizations may lose critical opportunities for change. The corresponding interview transcripts are as follows.

*“Although we are not their supplier of the whole machine project*, *we can go for the mechanical parts project of this whole machine after the whole machine is imported*. *This is our first step (A19—strategic adjustment)*.*” (YSTJ-645)*.

*“Although we have no factories and may not have advantages in capacity supply chain*, *we are asset-light operation and have certain organizational flexibility*, *which makes ‘a small ship makes a good turn’*. *To get bigger and stronger in this industry*, *we think it is inevitable that [companies] will go vertically integrated*, *but we also need time and adjust gradually (A3—change leading)*.*” (PPKW-151)*.

#### Integration for boundary-spanning

Integration for boundary-spanning refers to the capability of TMT through scouting, coordinating, and gathering internal and external resources to establish a cooperative community and promote the realization of organizational goals. Trans-boundary integration also includes horizontal integration within the TMT and vertical integration at all levels of the organization. The corresponding interview transcripts are as follows.

*“We compete in national and even international competitions*, *and we go every year to…*. *In this process*, *we will look for opportunities to cooperate with relevant international and domestic enterprises and integrate resources (A1—boundary integration)*. *… In the whole process*, *if you want to integrate external resources*, *you must first have your own value*, *so that you can integrate your own resources by exchanging them with others (A15—collaborative promotion)” (GHWL-379)*.

#### Resilience for boundary-spanning

As the saying goes, “natural selection, survival of the fittest”; RBS refers to the resilience of the TMT leading the organization to adapt to change in the uncertain environment. This is also supported by the analysis of coding interview transcripts.

*“Now in the 21st century…*. *You may not have an office*, *and we may not even have time to see each other*, *but we can still work in a company*. *Respect for individuality is the premise of what we call boundary-spanning cooperation*. *Starting a company is also a kind of cooperation*, *especially when getting into the operation stage (A16—circumstance adjustment) … why should we expect the 80s and 90s generation to work step-by-step? If they are treated with more respect and tolerance*, *their innovation and enthusiasm can be stimulated…*. *This kind of culture can gradually blend into the blood of the organization and help the organization to go further (A2—boundary reconstruction)*.*” (YSTJ-758)*.

The above analysis suggests that TMTBSL involves dynamic, interactive process among individuals in groups especially for TMTs (Pearce and Conger, 2003). The results of the qualitative study reveals that leadership may be seen as a group characteristic and a web of influence covering the groups under study, and that boundary-spanning leadership practices involve clarifying and valuing differences (roles, purpose, or expertise, for example) across boundaries in ways that build safety and respect – and then bringing different groups together to achieve a larger purpose (Cross et al., 2015). In summary, TMTBSL can be defined as the combined influence emerging from organizational strategy decision-making teams to carry out organizational cross-boundary innovation.

### Scale development

#### Item generation

Referring to the procedure of scale development (Farh et al., 1997; Carpenter, 2018), we initially compiled 35 descriptive statements containing the coded concept nodes, in which each dimension includes at least three items. All members of the research team participated in the discussion to ensure whether the descriptions appropriately covered the content, and if the expression was clear and concise in Chinese. Three professors of our research team read the complete set of responses individually. They eliminated those statements that were leadership behaviors or traits of a single leader, and those have the issues of ambiguous language and poor readability. After this round, 25 items were preserved.

In addition, a content evaluation assessment by six senior managers and eight middle managers (64.3% were male) from two high-tech enterprises interviewed previously was conducted. In this section, we designed a questionnaire asking for the retention and expression optimization of the items. To be specific, in order to avoid neutral answer, we designed four options (i.e., very unimportant, unimportant, important, and very important) to collect the importance of each item. We also considered experts’ comments and suggestions on how to modify items. According to their feedback, the items were revised again and five of them were deleted due to information redundancy and conceptualization misfit (Tang et al., 2017). Finally, we developed a 20-item TMTBSL scale.

#### Exploratory factor analysis

We employed EFA to examine the structure of the 5-dimension 20-item scale, ranging from 1 (“completely disagree”) to 5 (“completely agree”). The samples used in EFA were employees from a listed manufacturing company in Southwest China. We distributed 300 questionnaires and 227 participants returned the questionnaires, with a 210 of them were valid. Among the participants, approximately 59% were male, with 50.5% under the age of 40 years, 71.9% with organizational tenures of less than 6 years, and 24.2% in managerial positions (5.2% in senior and middle positions and 19% in junior positions).

The fitness test of the data showed that the Kaiser–Meyer–Olkin value was 0.949, indicating that the data were suitable for factor analysis. Bartlett’s test of sphericity revealed the moderation of the correlation matrix between variables (χ^2^ = 3585.655, *p* < 0.001), indicating that there are common factors between variables, thereby implying suitability for factor analysis. According to the rotary component matrix analysis results (i.e., principal component approach), we deleted items with a loading higher than 0.5 on both factors (Roesch and Rowley, 2005). Followed by Farh et al. (1997), a 14-item scale of TMTBSL was obtained after selecting and retaining the three items with the highest factor loading coefficient. The orthogonal maximum rotation method was selected again for EFA (see Table 2). The Cronbach’s alpha coefficient of the total scale was 0.975 and all five factors were above 0.70, indicating an acceptable reliability. As suggested by Carpenter (2018), we used the focus group approach to review the meanings and wording of the items to ensure the basic conceptualization and dimensional content validity of TMTBSL (Clark and Watson, 1995; Worthington and Whittaker, 2006).

**Table 2:** Exploratory factor analysis for TMTBSL.

Measurement items	*Cronbach’s α*	Factor loading				
		1	2	3	4	5
Foresight for boundary-spanning	0.933					
The current performance of the organization benefits from [their] integration of resources years ago		0.808				
The current accumulation of organizational advantage resources benefits from [their] assertive determination years ago		0.783				
The current strategy of organization is benefited from [their] understanding of macro policy years ago		0.674				
Resilience for boundary-spanning	0.924					
To encourage transformation and innovation, [they] value building diverse and inclusive organizational cultures			0.735			
To adapt to the changing environment, [they] always seize the opportunity to adjust the organizational strategy			0.718			
To maintain competitiveness, [they] encourage to build flexible boundaries (in business, product, etc.)			0.650			
Inspiration for boundary-spanning	0.916					
[They] show people what to strive for and where to go, so people are attracted to follow				0.742		
[They] have some personal charisma that attracts people to follow				0.715		
[They] have strong business skills and confidence in achieving organizational goals that attracts people to follow				0.632		
Integration for boundary-spanning	0.943					
[They] value communication between different departments and integration of information resources					0.717	
[They] value the process of strategy communication to make sure each member of the organization has the same goals					0.660	
[They] value collaboration to realize the integration of business and social resources					0.646	
Decisiveness boundary-spanning	0.923					
At the critical time when market conditions and policies are positive, [they] often take decisive developmental decisions						0.708
In the face of difficulty or crisis, [they] usually speak up rather than run away in the first place						0.576
Percentage of variance explained (86.809% in total)	0.975	22.136	19.234	19.214	17.240	11.471

*N* = 210. The factor analysis was based on principle components analysis and varimax rotation. The directive-achieving items are translated from Chinese.

To further examine the validity of the scale, we adopted 27% grouping criteria for the item analysis through the standards followed by Churchill (1979). That is, the (a) correlations between the items and total, (b) Cronbach’s alpha coefficient after deletion, and (c) discriminant degree composite reliability (CR). The results showed that the correlations between items and the total, and the discrimination degree CR both reached 0.001 significance, and the Cronbach’s alpha coefficient of the total table was less than 0.975 regardless of which item was deleted, indicating that the 14-item scale of TMTBSL has good validity.

#### Confirmatory factor analysis

The CFA was conducted to examine the structure of the scale, which was measured using a 5-point Likert scale ranging from 1 (“completely disagree”) to 5 (“completely agree”). Two sources of data were collected to control common method biases (Hinkin, 1995; Podsakoff et al., 2003). We used both online and offline approaches to distinguish the source of the samples. The online approach, using the snowball investigation and point-to-point methods, involved distributing and collecting 250 questionnaires. The offline approach involved another 250 questionnaires in an organization where we had conducted interviews before. In total, 148 responses were received and 144 were valid. In sum, a total of 500 questionnaires were distributed, 398 were recovered and 394 were valid. Approximately 46.7% of the participants were male, 95.5% under the age of 40 years, 74.2% with organizational tenures of less than 6 years, and 62.2% in managerial positions (23.1% in senior and middle positions and 39.1% in junior positions); 27.2% of them were from the state-owned organizations.

To test the model fitness effect of TMTBSL, we employed a first-order five-factor model of TMTBSL as the benchmark model, which was combined with the internal relationship among the five factors. Then, we tested the fit of the second-order model and compared it with the alternative models (Jarvis et al., 2003). As shown in Table 3, the first-order one-factor model and the second-order five-factor model are mathematically equivalent. The data analysis results are shown in Table 3.

**Table 3:** Confirmatory factor analysis for TMTBSL.

Models	χ^2^	df	Δχ^2^	χ^2^/df	TLI	CFI	NFI	RMSEA
Second-order, five-factor model	136.18	72		1.891	0.951	0.961	0.922	0.048
First-order, five-factor model	127.52	67		1.903	0.951	0.964	0.927	0.048
First-order, four-factor model	177.39	71	49.87^***^	2.498	0.918	0.936	0.899	0.062
First-order, three-factor model	214.53	74	87.01^***^	2.899	0.896	0.916	0.878	0.070
First-order, two-factor model	231.65	76	104.13^***^	3.048	0.888	0.906	0.868	0.072
First-order, one-factor model	253.78	77	126.26^***^	3.296	0.874	0.894	0.855	0.076

*N* = 394.

*** *p* < 0.001.

First-order, four-factor model: FBS + RBS; First-order, three-factor model: FBS + ItBS + RBS; First-order, two-factor model: FBS + IsBS + DBS, RBS + ItBS.

As indicated in Table 3, the fitness of the first-order five-factor model was tested (χ^2^/df = 1.903, RMSEA = 0.048, CFI, NFI, TLI > 0.90). Compared with the fitting test results of the other four models, the first-order five-factor model proved to be the best model among the first-order models. In addition, the fitness of the second-order five-factor model (χ^2^/df = 1.891, RMSEA = 0.048, CFI, NFI, TLI > 0.90) was good, too, indicating that TMTBSL should better be a second-order structure composed of five first-order factors.

According to Fornell and Larcker’s (1981) suggestion, we examined the CR and average variance extracted (AVE) of the scale to assess the construct validity, aggregate validity, and discriminant validity. Table 4 reveals that the CR values were between 0.814 and 0.852, higher than the standard of 0.70 (Hair et al., 2010). The AVE values were between 0.594 and 0.716. In addition, the factor loading corresponding to each dimension was greater than 0.7. Discriminant validity refers to the degree of difference between one construct and the others, which can be compared and judged by the square root of AVE and the absolute value of correlation coefficients of each latent variable. As shown in Table 4, AVE square root of each dimension is higher than the correlation coefficient with other dimensions. Taken together, these results demonstrate good construct validity, aggregate validity, and discriminant validity.

**Table 4:** Means, standard deviations, scale reliabilities, correlations, CR, and AVE for TMTBSL.

	*M*	*SD*	1	2	3	4	5
1. FBS	3.959	0.683	(0.810)				
2. IsBS	3.886	0.751	0.586^***^	(0.802)			
3. DBS	3.791	0.778	0.432^***^	0.526^***^	(0.846)		
4. ItBS	3.972	0.700	0.511^***^	0.474^***^	0.460^***^	(0.771)	
5. RBS	3.812	0.757	0.517^***^	0.552^***^	0.481^***^	0.467^***^	(0.784)
Cronbach’s *α*			0.933	0.924	0.916	0.943	0.923
CR			0.852	0.814	0.835	0.844	0.828
AVE			0.657	0.594	0.716	0.643	0.615

*N* = 394.

*** *p* < 0.001.

FBS, foresight for boundary-spanning; IsBS, inspiration for boundary-spanning; DBS, decisiveness for boundary-spanning; ItBS, integration for boundary-spanning; RBS, resilience boundary-spanning. CR, composite reliability; AVE, average variance extracted. The square roots of AVE are shown on the diagonal.

## Study 2: Top management team boundary-spanning leadership and innovative behavior

With the increased complexity of technical, products and services, organizational performance largely depends on employees’ ability to collaborate across various kinds of boundaries: vertical, horizontal, stakeholder, and geographic (Cross et al., 2015). When an organization is looking to spur innovation, the employees who could provide the broadest performance benefit to the organization struggled the most with this kind of collaboration. Innovative behavior is a multi-stage process of problem recognition, generation of ideas or solutions, building support for ideas, and idea implementation (Scott and Bruce, 1994). However, most importantly, organizational collaboration and innovation is not spontaneous, but requires its own intervention such as some key leadership (Cross et al., 2015).

Research found that the stimulation to innovate comes from the inspiring nature of the climate. Numerous research has shown support for the organizational climate-innovative behavior link (Scott and Bruce, 1994; Madrid et al., 2014; Wallace et al., 2016). Besides, team-related factors can also affect individual innovation behaviors, such as leader-member exchange (Scott and Bruce, 1994), team reflexivity (Litchfield et al., 2018). Other studies have explored how narcissistic (Wisse et al., 2015) or problem-solving style (Scott and Bruce, 1994) promote individual innovative behavior from the perspective of individual traits or styles. However, more studies have explored how leadership promotes individual innovative behavior, especially transformational leadership (Pieterse et al., 2010; Aryee et al., 2012) and entrepreneurial leadership (Newman et al., 2018; Bagheri et al., 2020).

Study 1 enables us to comprehensively understand boundary spanning leadership. That is, TMTBSL is probably a kind of the plural forms of leadership (Denis et al., 2012). And the results support Gibb’s view that “Leadership is probably best conceived as a group quality, as a set of functions which must be carried out by the group (Gibb, 1954, p. 884).” These understandings extend the conception of leadership beyond the idea that leadership can be shared between specific individuals to encompass possible reformulations of the notion of leadership itself as constituted by collective processes and interactions (Denis et al., 2012).

Previous studies have shown that various leadership has great impacts on organizational innovation and employee innovative behavior (Scott and Bruce, 1994; Tierney et al., 1999; Choi et al., 2016), especially considering some certain behaviors or traits of the direct leaders. However, most research tends to ignore the hierarchy of the leader, that is, to assume that the leading process and effects are the same for both leaders at the top or bottom (Zaccaro and Horn, 2003). Organizations are hierarchical structures, and the TMTs cannot directly affect every employee unless concerning some certain paths and ways. In addition, it should be noted that not all organizational members have the opportunity to directly interact with the TMTs. However, there is no denying that the TMTs can exert influence by inspiring the achievement goals to the organizational members (Nerstad et al., 2013). In addition, compared with lower-level leaders, senior leaders have the advantages in obtaining and controlling more organizational information and resources, and can exert greater and far-reaching influence through making strategic decisions to the organization (Ernst and Chrobot-Mason, 2011). As argued above, we are interested in exploring the path through which TMTBSL influence employees’ innovative behavior through the perceived motivational climate.

### Theoretical background and hypotheses

#### Top management team boundary-spanning leadership and employee innovative behavior

Innovative behavior refers to the intentional generation, promotion, and realization of new ideas within a work role, workgroup, or organization (Scott and Bruce, 1994; Miron-Spektor and Beenen, 2015). Leadership has proved to be one of the key factors in facilitating employee innovative behavior (Wang et al., 2019) given the circumstances that leadership may help facilitate interaction and information exchange across group boundaries (Harvey and Edmondson, 2016). A large number of studies have confirmed the impact of different leadership styles on employee innovative behavior (e.g., Graham, 2013; Salem et al., 2018).

Through the lens of social information processing theory (SIPT), individuals’ attitudes and behaviors are not only determined by their own needs and goals but also greatly influenced by the prevailing social environment (Salancik and Pfeffer, 1978). As the important information sources in the workplace, TMTs are the most important leaders to make changes and promote boundary-spanning behavior at the strategic level (Morgeson et al., 2010; Liu et al., 2021). As illustrated by Study 1, it is committed to the realization of an organization’s visions and goals. Previous studies have demonstrated that boundary-spanning behavior of leaders can help employees acquire valuable knowledge by expanding their knowledge search (Takanashi and Kyoung-Joo, 2018), which is a typical innovative behavior (Katila and Ahuja, 2002). Therefore, we propose the following:

*Hypothesis 1*: TMTBSL has a positive effect on employee innovative behavior.

#### The mediating role of a motivational climate

Perceived work motivational climate refers to employees’ perceptions of the extant criteria of success and failure (Nerstad et al., 2013). It can help employees understand what they can do to achieve success at work or recognition by the organization. Perceived motivational climate has two dimensions: mastery (or task-involving) climate and performance (or ego-involving) climate. The mastery climate refers to an individual’s perception that demonstrated effort, sharing, and cooperation are valued, and the performance climate focuses on achieving outcomes and normative competence (Ntoumanis and Biddle, 1999).

According to SIPT, as an information source which describes the characteristics of the corresponding work environment in the social environment (Salancik and Pfeffer, 1978), TMTBSL can promote employees’ perception of motivational climate and provide a goal-oriented environment for them (Nerstad et al., 2013). Specifically, TMTBSL encourages employees to integrate resources through boundary-spanning and establish cooperative communities to realize resource sharing and enhance the mastery climate in organizations (Černe et al., 2014). Furthermore, TMTBSL focuses on the adaptability and maintenance ability of an organization in an uncertain environment, and advocates creating high performance climate to enhance the competitiveness and vitality of an organization (Černe, 2012). We hypothesized that TMTBSL may enhance organizational employees’ perception of motivational climate by promoting the formation of mastery climate and performance climate. Therefore, we propose the following:

Hypothesis 2: TMTBSL has a positive effect on perceived motivational climate.

Innovative behavior is a requirement for many jobs today, for it involves actions such as seeking out new ideas, championing new initiatives, and securing planning for implementation.. With the increase of environmental uncertainty and complexity, organizations have been increasingly relying on employees’ innovation (Chen et al., 2016). However, innovation is also a kind of activity with a high risk of failure (van der Panne et al., 2003). Thus, leaders’ identification and implementation about change may be an important factor to employee innovative behavior. According to SIPT, when faced with high uncertainty and complexity, individuals process and interpret specific social information to decide what attitude and behavior to adopt (Salancik and Pfeffer, 1978). As the goal orientation of the environment in which employees are located, perceived motivational climate is likely to affect the decision of whether employees will conduct innovative behavior. Therefore, we propose the following:

*Hypothesis 3*: Perceived motivation climate positively affects employee innovative behavior.

Top management team boundary-spanning leadership is able to integrate organizational goals and visions into followers’ goals, encourage employees to transcend the scope of previous work by creating high expectations and visions, and motivate them to solve problems in innovative ways to achieve innovation (García-Morales et al., 2012). Studies have showed that the influence of leadership and work group on innovative behavior requires a certain kind of climate for innovation (e.g., psychological climate, Scott and Bruce, 1994). Meanwhile, as the most critical source of information in the workplace, the influence of TMTs plays a very important role in stimulating employee innovative behavior (Yu and Frenkel, 2013; Miron-Spektor and Beenen, 2015). Therefore, we propose the following:

*Hypothesis 4*: Perceived motivational climate mediates the relationship between TMTBSL and employee innovative behavior.

**Figure 2:**
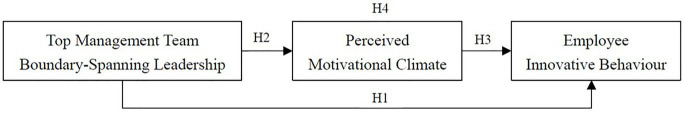
Proposed conceptual model.

Figure 2 demonstrates the proposed conceptual model and the hypotheses discussed in this section.

### Method

#### Measurements

The data of Study 2 were collected through a questionnaire survey. A 5-point Likert scale, ranging from 1 (“completely disagree”) to 5 (“completely agree”), was used to measure the main variables. The TMTBSL scale is the 5-dimension 14-item scale in Chinese developed in Study 1. The innovative behavior scale is a revised 8-item scale in Chinese followed by Zhou (2003). The perceived motivational climate scale is a 2-dimension 14-item scale developed by Nerstad et al. (2013) and we followed the back-translation method to translate it into Chinese (Brislin, 1986).

#### Samples

A total number of 504 valid questionnaires were collected and 151 of them were from a listed company located in North China, a newly diversified food processing enterprise, and the other 353 were from several state-owned and private organizations in Southwest China. Approximately 59.3% of the participants were male. Majority of the participants were younger than 40 years old (79.8%). 39.9% of them were in managerial positions (17.3% in senior and middle positions and 22.6% in junior positions). In addition, 48.8% of them were from the state-owned organizations.

### Results

#### Confirmatory factor analysis

The reliability test results of this paper show that the Cronbach’s alpha coefficients of TMTBSL, perceived motivational climate, and innovative behavior are 0.978, 0.936, and 0.939, respectively. The three-factor model which showed superior model fit than other alternative models: χ^2^ (21) = 43.869, χ^2^/*df* = 2.675, RMSEA = 0.047, CFI = 0.995, TLI = 0.992. Therefore, the variables involved in Study 2 displayed representatively different concepts, and the measure has good discriminative validity. The CFA results are shown in Table 5.

**Table 5:** Comparison of alternative measurement models.

Models	Factors	χ^2^	df	Δχ^2^	χ^2^/df	RMSEA	CFI	TLI
1	*Three factors*: TMTBSL, PMC, IB	43.869	21		2.675	0.047	0.995	0.992
2	*Two factors*: TMTBSL+PMC, IB	209.957	26	166.088^***^	8.075	0.119	0.961	0.946
3	*Two factors*: TMTBSL, PMC + IB	305.358	26	261.489^***^	11.745	0.146	0.940	0.918
4	*Two factors*: TMTBSL+IB, PMC	498.433	26	454.564^***^	19.171	0.190	0.899	0.861
5	*One factor*: TMTBSL+PMC + IB	515.332	27	471.463^***^	19.086	0.190	0.896	0.861

*N* = 504.

*** *p* < 0.001.

TMTBSL, top management team boundary-spanning leadership; PMC, perceived motivational climate; IB, innovative behavior.

#### Correlation analysis

The correlation analysis results of all variables are shown in Table 6, and the mean values of all variables are basically consistent with reality. In addition, there were positive correlations between TMTBSL and employee innovative behavior (*r* = 0.629, *p* < 0.001), TMTBSL and perceived motivational climate (*r* = 0.751, *p* < 0.001), and perceived motivational climate and employee innovative behavior (*r* = 0.677, *p* < 0.001).

**Table 6:** Means, standard deviations, scale reliabilities and correlations of all variables involved in Study 2.

Variables	Mean	*SD*	1	2	3	4	5	6	7	8	9
1. Gender	1.407	0.492									
2. Age	3.367	1.414	−0.230^**^								
3. Education level	1.573	0.587	0.113^*^	−0.137^**^							
4. Working years	8.37	9.714	−0.139^**^	0.456^**^	−0.018						
5. Position level	3.39	0.849	0.172^**^	−0.199^**^	−0.244^**^	−0.135^**^					
6. Organization type	3.282	1.696	0.106^*^	−0.110^*^	0.217^**^	0.029	0.057				
7. TMTBSL	3.972	0.74	−0.140^**^	−0.003	−0.139^**^	−0.005	−0.111^*^	−0.040	0.978		
8. PMC	3.878	0.675	−0.185^**^	0.033	−0.149^**^	0.035	−0.084	0.000	0.751^**^	0.936	
9. IB	4.004	0.64	−0.120^**^	−0.034	−0.047	0.036	−0.082	0.044	0.629^**^	0.677^**^	0.939

*N* = 504. Cronbach’s alphas are shown on the diagonal.

^**^p < 0.01; ^***^p < 0.001.

TMTBSL, top management team boundary-spanning leadership; PMC, perceived motivational climate; IB, innovative behavior.

#### Analysis

The SPSS.21 software was used in Study 2 to run the analyses. The results are shown in Table 7. As shown, TMTBSL had significant positive effects on perceived motivational climate (M2, *β* = 0.673, *p* < 0.001) and innovative behavior (M4, β = 0.542, *p* < 0.001). Hypotheses 1 and 2 were supported. Perceived motivational climate and innovative behavior (M5, *β* = 0.638, *p* < 0.001) had significant positive effects. Additionally, it also confirmed the mediating effect of perceived motivational climate (M6, β = 0.253, *p* < 0.001). These results support Hypotheses 3 and 4. Furthermore, to test the mediating effect of perceived motivational climate (Hayes and Scharkow, 2013), the upper and lower limits of the bootstrap of the direct effect, indirect effect, and total effect were not 0, indicating that the direct effect of TMTBSL (Boot SE = 0.042, [0.170, 0.337]), indirect effect of perceived motivational climate (Boot SE = 0.051, [0.196, 0.396]), and total effect (Boot SE = 0.031, [0.482, 0.602]) exists.

**Table 7:** Hierarchy regression results of all variables in Study 2.

Variables	Perceived Motivational Climate		Innovative behavior			
	M1	M2	M3	M4	M5	M6
Intercept	4.947	1.454	4.789	1.975	1.634	1.352
Control variables						
1. Gender	−0.233^***^	−0.117^***^	−0.169^***^	−0.075	−0.020	−0.025
2. Age	−0.012	0.015	−0.034	−0.012	−0.027	−0.019
3. Education level	−0.222^***^	−0.077^*^	−0.114^*^	0.003	0.028	0.036
4. Organization type	−0.002	−0.006	−0.002	−0.006	−0.001	−0.003
5. Department type	−0.006	−0.015	0.020	0.013	0.024	0.020
6. Position level	−0.096^*^	0.003	−0.092^*^	−0.012	−0.031	−0.013
7. Working years	0.000	0.000	0.002	0.002	0.003	0.003
8. Years of working with leaders	0.002^*^	0.002^***^	0.001	0.002^***^	0.000	0.001
9. Income	−0.061	−0.047	−0.071	−0.060	−0.032	−0.040
Independent variables						
10. TMTBSL		0.673^***^		0.542^***^		0.253^***^
11. PMC					0.638^***^	0.429^***^
*R^2^*	0.077	0.589	0.052	0.422	0.470	0.506
*F*	4.577	70.590	2.983	35.935	43.665	45.769

*N =* 504.

^*^p < 0.01; ^***^p < 0.001.

TMTBSL, top management team boundary-spanning leadership; PMC, perceived motivational climate; IB, innovative behavior.

### Discussion

#### Conclusion

This research focused on the concept construction and scale development of TMTBSL, as well as its influence on innovative behavior. Through biographical data analysis and semi-structured interviews, we found that TMTBSL is not a simple stack of the boundary-spanning leadership of single leaders in TMT (Raes et al., 2007), but the combined influence of the leadership process. Moreover, TMTBSL is more than behaviors related to boundary management or some specific identity such as that of an outside director. By using classic grounded theory, the five dimensions of TMTBSL have been extracted. Following the procedure of scale development (Farh et al., 1997; Carpenter, 2018), and the constructive cognition of classical grounded theory, a 5-dimension 14-item scale of TMTBSL was developed with good reliability and validity according to the results of EFA and CFA. Furthermore, the empirical results of Study 2 showed that TMTBSL has a positive impact on employee innovative behavior *via* perceived motivational climate.

#### Theoretical contribution

The findings of this study provide comprehensive understanding for the connotation of TMTBSL. Instead of discussing individual-level boundary-spanning leadership or related behaviors (Salem et al., 2018) or the demographics of the TMTs (Finkelstein and Hambrick, 1990), we framed TMTBSL as collective leadership or influence inside and outside organizations. Existing research on boundary-spanning leadership mainly focuses on single leaders and explains their behavioral process of single leaders with boundary-spanning behaviors, thereby failing to reveal the specific connotation of strategic TMTBSL from the perspective of senior leader teams. This research points out that the core connotation of TMTBSL is not just the behaviors or influences displayed by individual leaders, but a complex resultant force formed by the interaction of numerous related influences. The five dimensions of TMTBSL reflect its process of emergence.

Second, this study develops a measurement tool of TMTBSL. By doing so, we provide novel information to the TMTBSL literature. Existing scales consist of relatively unidimensional indicators such as serving as an outside director (Ferguson et al., 2019) or boundary-spanning leadership for expatriates (Salem et al., 2018) and focus on the explicit boundaries across organizations. We defined the boundaries in organizations based on previous literature in Study 1 (Ancona and Caldwell, 1992; Ernst and Chrobot-Mason, 2011; Cross et al., 2013), and developed a scale to measure TMTBSL. Consequently, we provide operational tool for future studies.

Furthermore, the definition and scale development of TMTBSL allows us to establish and examine how TMTBSL is perceived by employees and promotes individual innovation. Arguably, if organizational changes are not recognized by employees, organizational strategies will be difficult to implement. Hence, the results of our research reveal the multiple roles of TMTBSL and highlight how it positively affects the innovative behavior of organizational members through the lens of social information processing by discussing the mediating role of motivational climate.

#### Practical implications

There is increasing calling that organizations need to cross boundaries, but not just boundaries of their internal functions and responsibilities or those of different industries. The BSL which demonstrated by a single leader or mid-level leader is more about interpersonal collaboration and resource integration. As a matter of fact, such boundary-spanning activities carried out by non-strategic leaders are unable to spur in-depth reform and innovation to reach the essence of organization. Therefore, the role of TMTBSL for organizational change and development become increasingly prominent. Virtuous cycle development in an organization should be accomplished by working from an organizational strategy level, allowing TMTs to engineer innovation at a core level, and demonstrating TMTBSL to influence the cross-boundary innovation at all levels of the organization. The investigation of the concept, dimensions, and scale of TMTBSL are also conducive to the selection or cultivation of organizational successors. It has important practical value for the development of leadership. The types of leadership suitable for organizational development are constantly evolving with the times.

#### Limitations and future research

When extracting the items and dimensions of TMTBSL, we mainly adopted the method of rooted coding, which is inevitably affected by subjective experience. Future research can supplement more supportive cases, texts, or interview materials from different sources, cultural backgrounds, or organizational attributes. Although the data used in developing the TMTBSL scale in this research were collected from different sources, they may suffer from a lack of interpretation in other cultural backgrounds. We encourage future study to collect data from different cultural backgrounds to gain a cross-cultural validity of TMTBSL. Finally, future studies are recommended to explore the mechanisms of TMTBSL from different theoretical perspectives.

## Data availability statement

The original contributions presented in the study are included in the article/supplementary material, further inquiries can be directed to the corresponding author.

## Author contributions

LXM proposed the research framework and conducted the coding analysis. YY designed the coding process and analyzed data. ZXW conducted the literature research and reviewed manuscript. ZNJ revised and edited the manuscript. All authors discussed, finalized, and approved the manuscript for publication.

## Funding

This work was supported by the National Social Science Fund of China (Project No. 20VYJ016), Innovational Fund of Xihua University Postgraduate (Project No. YCJJ2020006), Institute of International Economics and Management Innovation Program (Project No. 20200013), and Open Project of Sichuan Applied Psychology Research Center (Project No. CSXL-212A05).

## Conflict of interest

The authors declare that the research was conducted in the absence of any commercial or financial relationships that could be construed as a potential conflict of interest.

## Publisher’s note

All claims expressed in this article are solely those of the authors and do not necessarily represent those of their affiliated organizations, or those of the publisher, the editors and the reviewers. Any product that may be evaluated in this article, or claim that may be made by its manufacturer, is not guaranteed or endorsed by the publisher.

## Footnotes

^1^Hurun Report (2021). Hurun China rich list 2021. https://www.hurun.net/zh-CN/Rank/HsRankDetails?pagetype=rich (Accessed February 9, 2022).
