# Supplementary material for: Top management team boundary-spanning leadership: Measurement development and its impact on innovative behavior
Source: Front Psychol. 2022 Dec 15;13:988771. doi: 10.3389/fpsyg.2022.988771 (PMC9798318; doi:10.3389/fpsyg.2022.988771)
Supplement: Supplementary file 1 [file Data_Sheet_1.docx]

# **Tables**

**Table 1.** Coding results of TMTBSL

| **Dimensions** | **Subdimensions** | **Concept nodes (Partial)** |
| --- | --- | --- |
| IsBS |  |  |
|  | Vision inspiring | （a18）establish vision and goals that employees generally agree on; … |
|  | Trait influence | （a12）attract people inside and outside the organisation with virtu; … |
|  | Capability attraction | （a1）has the ability to lead and achieve goals; … |
| DBS |  |  |
|  | Responsibility taking | （a35）take responsibility and has courage to make decisions; … |
|  | Strategy adjustment | （a26）choose the right strategy to promote the healthy development of the organisation; … |
|  | Change leading | （a42）lead transformation across boundaries; … |
| FBS |  |  |
|  | Insight perception | （a55）has a keen sense of timing and seize opportunities; … |
|  | Perspective forecast | （a76）pay attention to the development prospect and grasp the right direction; … |
|  | Boundary-spanning [mindset](javascript:;) | （a74）has ground-breaking thinking beyond normal; … |
| ItBS |  |  |
|  | Boundary integration | （a102）reach goals across geographical boundaries; … |
|  | Consensus building | （a98）collaborate across boundaries to achieve common goals; … |
|  | Collaborative promotion | （a89）good at cross-boundary joint innovation; … |
| RBS |  |  |
|  | Diversity inclusive | （a124）good at creating an inclusive corporate culture; … |
|  | Circumstance adjustment | （a140）adapt to local conditions and carry out cross-boundary operations; … |
|  | Boundary reconstruction | （a143）build strategic alliances and realise resource sharing; … |

Note：The author collates the results according to the coding results. The above concept nodes are all translated from Chinese.

FBS = Foresight for Boundary-Spanning; IsBS = Inspiration for Boundary-Spanning; DBS = Decisiveness for Boundary-Spanning; ItBS = Integration for Boundary-Spanning; RBS = Resilience Boundary-Spanning.

**Table 2.** Exploratory factor analysis for TMTBSL

| **Measurement items** | ***Cronbach's α*** | **Factor loading** | | | | |
| --- | --- | --- | --- | --- | --- | --- |
|  |  | **1** | **2** | **3** | **4** | **5** |
| Foresight for Boundary-Spanning | 0.933 |  |  |  |  |  |
| The current performance of the organisation benefits from [their] integration of resources years ago |  | 0.808 |  |  |  |  |
| The current accumulation of organisational advantage resources benefits from [their] assertive determination years ago |  | 0.783 |  |  |  |  |
| The current strategy of organisation is benefited from [their] understanding of macro policy years ago |  | 0.674 |  |  |  |  |
| Resilience for Boundary-Spanning | 0.924 |  |  |  |  |  |
| To encourage transformation and innovation, [they] value building diverse and inclusive organisational cultures |  |  | 0.735 |  |  |  |
| To adapt to the changing environment，[they] always seize the opportunity to adjust the organisational strategy |  |  | 0.718 |  |  |  |
| To maintain competitiveness, [they] encourage to build flexible boundaries (in business, product, etc.) |  |  | 0.650 |  |  |  |
| Inspiration for Boundary-Spanning | 0.916 |  |  |  |  |  |
| [They] show people what to strive for and where to go, so people are attracted to follow |  |  |  | 0.742 |  |  |
| [They] have some personal charisma that attracts people to follow |  |  |  | 0.715 |  |  |
| [They] have strong business skills and confidence in achieving organisational goals that attracts people to follow |  |  |  | 0.632 |  |  |
| Integration for Boundary-Spanning | 0.943 |  |  |  |  |  |
| [They] value communication between different departments and integration of information resources |  |  |  |  | 0.717 |  |
| [They] value the process of strategy communication to make sure each member of the organisation has the same goals |  |  |  |  | 0.660 |  |
| [They] value collaboration to realise the integration of business and social resources |  |  |  |  | 0.646 |  |
| Decisiveness Boundary-Spanning | 0.923 |  |  |  |  |  |
| At the critical time when market conditions and policies are positive, [they] often take decisive developmental decisions |  |  |  |  |  | 0.708 |
| In the face of difficulty or crisis, [they] usually speak up rather than run away in the first place |  |  |  |  |  | 0.576 |
| Percentage of variance explained（86.809% in total） | 0.975 | 22.136 | 19.234 | 19.214 | 17.240 | 11.471 |

*N* = 210. The factor analysis was based on principle components analysis and varimax rotation. The directive-achieving items are translated from Chinese.

**Table 3.** Confirmatory factor analysis for TMTBSL

| **Models** | ***χ^2^*** | ***df*** | ***Δχ^2^*** | ***χ^2^/df*** | **TLI** | **CFI** | **NFI** | **RMSEA** |
| --- | --- | --- | --- | --- | --- | --- | --- | --- |
| Second-order, five-factor model | 136.18 | 72 |  | 1.891 | 0.951 | 0.961 | 0.922 | 0.048 |
| First-order, five-factor model | 127.52 | 67 |  | 1.903 | 0.951 | 0.964 | 0.927 | 0.048 |
| First-order, four-factor model | 177.39 | 71 | 49.87^***^ | 2.498 | 0.918 | 0.936 | 0.899 | 0.062 |
| First-order, three-factor model | 214.53 | 74 | 87.01^***^ | 2.899 | 0.896 | 0.916 | 0.878 | 0.070 |
| First-order, two-factor model | 231.65 | 76 | 104.13^***^ | 3.048 | 0.888 | 0.906 | 0.868 | 0.072 |
| First-order, one-factor model | 253.78 | 77 | 126.26^***^ | 3.296 | 0.874 | 0.894 | 0.855 | 0.076 |

*N* = 394.

^***^*p* < .001

First-order, four-factor model: FBS + RBS; First-order, three-factor model: FBS + ItBS + RBS; First-order, two-factor model: FBS + IsBS + DBS, RBS + ItBS.

**Table 4.** Means, standard deviations, scale reliabilities, correlations, CR and AVE for TMTBSL

|  | **M** | **SD** | **1** | **2** | **3** | **4** | **5** |
| --- | --- | --- | --- | --- | --- | --- | --- |
| 1. FBS | 3.959 | 0.683 | （0.810） |  |  |  |  |
| 2. IsBS | 3.886 | 0.751 | 0.586^***^ | （0.802） |  |  |  |
| 3. DBS | 3.791 | 0.778 | 0.432^***^ | 0.526^***^ | （0.846） |  |  |
| 4. ItBS | 3.972 | 0.700 | 0.511^***^ | 0.474^***^ | 0.460^***^ | （0.771） |  |
| 5. RBS | 3.812 | 0.757 | 0.517^***^ | 0.552^***^ | 0.481^***^ | 0.467^***^ | （0.784） |
| Cronbach’s *α* |  |  | 0.933 | 0.924 | 0.916 | 0.943 | 0.923 |
| CR |  |  | 0.852 | 0.814 | 0.835 | 0.844 | 0.828 |
| AVE |  |  | 0.657 | 0.594 | 0.716 | 0.643 | 0.615 |

*N* = 394.

^***^ *p* < .001

FBS = Foresight for Boundary-Spanning; IsBS = Inspiration for Boundary-Spanning; DBS = Decisiveness for Boundary-Spanning; ItBS = Integration for Boundary-Spanning; RBS = Resilience Boundary-Spanning.

CR = Composite reliability; AVE = average variance extracted.

The square roots of AVE are shown on the diagonal.

**Table 5.** Comparison of alternative measurement models

| **Models** | **Facotrs** | ***χ^2^*** | ***df*** | ***Δχ^2^*** | ***χ^2^/df*** | **RMSEA** | **CFI** | **TLI** |
| --- | --- | --- | --- | --- | --- | --- | --- | --- |
| 1 | *Three factors*: TMTBSL, PMC, IB | 43.869 | 21 |  | 2.675 | .047 | .995 | .992 |
| 2 | *Two factors*: TMTBSL+PMC, IB | 209.957 | 26 | 166.088^***^ | 8.075 | .119 | .961 | .946 |
| 3 | *Two factors*: TMTBSL, PMC+IB | 305.358 | 26 | 261.489^***^ | 11.745 | .146 | .940 | .918 |
| 4 | *Two factors*: TMTBSL+IB, PMC | 498.433 | 26 | 454.564^***^ | 19.171 | .190 | .899 | .861 |
| 5 | *One factor*: TMTBSL+PMC+IB | 515.332 | 27 | 471.463^***^ | 19.086 | .190 | .896 | .861 |

*N* = 504.

^***^ *p* < .001

TMTBSL = Top Management Team Boundary-Spanning Leadership; PMC = Perceived Motivational Climate; IB = Innovative behavior.

**Table 6.** Means, standard deviations, scale reliabilities and correlations of all variables involved in Study 2

| **Variables** | **Mean** | **SD** | **1** | **2** | **3** | **4** | **5** | **6** | **7** | **8** | **9** |
| --- | --- | --- | --- | --- | --- | --- | --- | --- | --- | --- | --- |
| 1. Gender | 1.407 | .492 |  |  |  |  |  |  |  |  |  |
| 2. Age | 3.367 | 1.414 | -.230^**^ |  |  |  |  |  |  |  |  |
| 3. Education level | 1.573 | .587 | .113^*^ | -.137^**^ |  |  |  |  |  |  |  |
| 4. Working years | 8.37 | 9.714 | -.139^**^ | .456^**^ | -.018 |  |  |  |  |  |  |
| 5. Position level | 3.39 | .849 | .172^**^ | -.199^**^ | -.244^**^ | -.135^**^ |  |  |  |  |  |
| 6. Organization type | 3.282 | 1.696 | .106^*^ | -.110^*^ | .217^**^ | .029 | .057 |  |  |  |  |
| 7. TMTBSL | 3.972 | .74 | -.140^**^ | -.003 | -.139^**^ | -.005 | -.111^*^ | -.040 | .978 |  |  |
| 8. PMC | 3.878 | .675 | -.185^**^ | .033 | -.149^**^ | .035 | -.084 | .000 | .751^**^ | .936 |  |
| 9. IB | 4.004 | .64 | -.120^**^ | -.034 | -.047 | .036 | -.082 | .044 | .629^**^ | .677^**^ | .939 |

*N* = 504. Cronbach’s alphas are shown on the diagonal.

^**^ *p* < .01; ^***^ *p* < .001

TMTBSL = Top Management Team Boundary-Spanning Leadership; PMC = Perceived Motivational Climate; IB = Innovative behavior.

**Table 7.** Hierarchy regression results of all variables in Study 2

| **Variables** | **Perceived Motivational Climate** | |  | | **Innovative behavior** | | | |
| --- | --- | --- | --- | --- | --- | --- | --- | --- |
|  | **M1** | **M2** |  | **M3** | | **M4** | **M5** | **M6** |
| Intercept | 4.947 | 1.454 |  | 4.789 | | 1.975 | 1.634 | 1.352 |
| Control variables |  |  |  |  | |  |  |  |
| 1. Gender | -0.233^***^ | -0.117^***^ |  | -0.169^***^ | | -0.075 | -0.020 | -0.025 |
| 2. Age | -0.012 | 0.015 |  | -0.034 | | -0.012 | -0.027 | -0.019 |
| 3. Education level | -0.222^***^ | -0.077^*^ |  | -0.114^*^ | | 0.003 | 0.028 | 0.036 |
| 4. Organization type | -0.002 | -0.006 |  | -0.002 | | -0.006 | -0.001 | -0.003 |
| 5. Department type | -0.006 | -0.015 |  | 0.020 | | 0.013 | 0.024 | 0.020 |
| 6. Position level | -0.096^*^ | 0.003 |  | -0.092^*^ | | -0.012 | -0.031 | -0.013 |
| 7. Working years | 0.000 | 0.000 |  | 0.002 | | 0.002 | 0.003 | 0.003 |
| 8. Years of working with leaders | 0.002^*^ | 0.002^***^ |  | 0.001 | | 0.002^***^ | 0.000 | 0.001 |
| 9. Income | -0.061 | -0.047 |  | -0.071 | | -0.060 | -0.032 | -0.040 |
| Independent variables |  |  |  |  | |  |  |  |
| 10. TMTBSL |  | 0.673^***^ |  |  | | 0.542^***^ |  | 0.253^***^ |
| 11. PMC |  |  |  |  | |  | 0.638^***^ | 0.429^***^ |
| *R^2^* | 0.077 | 0.589 |  | 0.052 | | 0.422 | 0.470 | 0.506 |
| *F* | 4.577 | 70.590 |  | 2.983 | | 35.935 | 43.665 | 45.769 |

*N* = 504.

^**^ *p* < .01; ^***^ *p* < .001

TMTBSL = Top Management Team Boundary-Spanning Leadership; PMC = Perceived Motivational Climate; IB = Innovative behavior.
