# Supplementary material for: Top management team boundary-spanning leadership: Measurement development and its impact on innovative behavior
Source: Front Psychol. 2022 Dec 15;13:988771. doi: 10.3389/fpsyg.2022.988771 (PMC9798318; doi:10.3389/fpsyg.2022.988771)
Supplement: Supplementary file 2 [file Table_1.DOCX]

**Appendix I**

**Biographical text information is used in this study**

| **NO.** | **Enterprises** | **Main industries** | **Book title** | **Number of words^a^** | **Publisher** | **Year of publication** |
| --- | --- | --- | --- | --- | --- | --- |
| 1 | Huawei Holdings Co. LTD | IT | *Huawei: The biography of Ren Zhengfei* | 319 | Sino-culture Press | 2016 |
| 2 | Baidu Online Network Technology Co., LTD | Search engine | *Robin Li: Focus on the achievements of Baidu life* | 182 | Taihai Publishing House | 2016 |
|  |  |  | *Creative Notes of Robin Li: The Treasure book of post-8090 entrepreneurship* | 244 | Chengdu Times Press | 2011 |
| 3 | Sf Express co., LTD | Logistics | *All the way SF Express: the legendary life of "King of express delivery"* | 208 | Beijing Times Chinese Press | 2016 |
| 4 | Hope Group Co. LTD | Agriculture, chemicals and finance | *The richest evergreen Liu Yongxing and Liu Yonghao: Hope Group for 30 years* | 250 | Central compilation & translation Press | 2010 |
|  | East hope Group Co. LTD | Agriculture and chemicals | *Chinese-style lean management（internal speech of Liu Yongxing）* | 275 | Guangdong Economy Publishing House | 2014 |
| 5 | Alibaba Network Technology Co. LTD | E-commerce and fintech | *This is Jack Ma* | 300 | Zhejiang People's Publishing House | 2015 |
|  |  |  | *Jack Ma (My management experience)* | 173 | Zhejiang People's Publishing House | 2017 |
| 6 | Tencent Technology (Shenzhen) Co., LTD | Internet service | *User-oriented (Ma Huateng's business management wisdom)* | 175 | Zhejiang People's Publishing House | 2018 |
| 7 | Xiaomi Technology Co. LTD | IT | *Lei Jun: stand on the tuyere* | 159 | Huazhong University of Science and Technology Press | 2013 |
| 8 | Evergrande Group Co LTD | Real estate and investment | *Evergrande Xu Jiayin: Suffering is my precious wealth* | 264 | Taihai Publishing House | 2017 |
| 9 | Giant Network Technology Co., LTD | Finance, online game and investment | *Shi Yuzhu's entrepreneurial wisdom* | 174 | Zhejiang University Press | 2010 |
| 10 | Beijing Qihoo Technology Co., LTD | Internet and search software | *The Disruptor: Zhou Hongyi's autobiography* | 365 | Beijing United Publishing Company | 2017 |
|  |  |  | *Rejecting mediocrity: Zhou Hongyi and his Legacy* | 213 | Citic Press | 2013 |
| 11 | Wahaha Group Co. LTD | beverage | *Zong Qinghou: The principle of gravitation* | 450 | Hongqi Press | 2015 |
| 12 | Lenovo Group Co. LTD | Computer services and investment | *Asked liu: Liu Chuanzhi's three elements of management* | 298 | Zhejiang People's Publishing House | 2015 |
|  |  |  | *Liu Chuanzhi Lenovo management diary* | 354 | China Railway Publishing House | 2011 |
| 13 | China Vanke Co., Ltd. | Real estate | *Management log of Wang Shi* | 305 | Zhejiang University Press | 2014 |
| 14 | Jingdong Group Co. LTD | E-commerce and finance | *Self-statement of Liu Qiangdong* | 136 | Citic Press | 2016 |
| 15 | Wanda Group Co. LTD | Real estate and cultural industry | *The richest man Wang Jianlin: a entrepreneur who has done great things* | 221 | Tianjin Renmin Press | 2017 |
| 16 | Hongta Group Co. LTD | Primary industry | *Management method of Chu Shijian* | 235 | China Friendship Publishing Company | 2016 |
|  |  |  | *Chu Shijian: entrepreneurs who influence entrepreneurs* | 320 | Hunan Literature and Art Publishing House | 2014 |

Note: The above information is based on the biographies selected for this study.

Number of words^a^: the measurement unit is a thousand of words.
